# Proteomic analysis of *Paracoccidioides brasiliensis* complex isolates: Correlation of the levels of differentially expressed proteins with *in vivo* virulence

**DOI:** 10.1371/journal.pone.0218013

**Published:** 2019-07-02

**Authors:** Cristiane Candida do Amaral, Geisa Ferreira Fernandes, Anderson Messias Rodrigues, Eva Burger, Zoilo Pires de Camargo

**Affiliations:** 1 Department of Medicine, Discipline of Infectious Diseases, Federal University of São Paulo (UNIFESP), São Paulo, Brazil; 2 Department of Microbiology, Immunology and Parasitology, Discipline of Cellular Biology, Federal University of São Paulo (UNIFESP), São Paulo, Brazil; 3 Department of Microbiology and Immunology, Federal University of Alfenas (UNIFAL), Alfenas, Brazil; University of South Africa, SOUTH AFRICA

## Abstract

**Background:**

Paracoccidioidomycosis (PCM) is a systemic mycosis commonly found in Latin America that is caused by distinct species of *Paracoccidioides* genus: *Paracoccidioides brasiliensis* complex (S1, PS2, PS3 and PS4) and *Paracoccidioides lutzii*. Its pathobiology has been recently explored by different approaches to clarify the mechanisms of host-pathogen interactions underpinning PCM. The diversity of clinical forms of this disease has been attributed to both host- and fungus-related factors.

**Methodology/Principal findings:**

For better understanding of the molecular underpinnings of host-fungus interactions, we evaluated *in vivo* virulence of nine *Paracoccidioides brasiliensis* complex isolates and correlated it to protein expression profiles obtained by two-dimensional gel electrophoresis. Based on the recovery of viable fungi from mouse organs, the isolates were classified as those having low, moderate, or high virulence. Highly virulent isolates overexpressed proteins related to adhesion process and stress response, probably indicating important roles of those fungal proteins in regulating the colonization capacity, survival, and ability to escape host immune system reaction. Moreover, highly virulent isolates exhibited enhanced expression of glycolytic pathway enzymes concomitantly with repressed expression of succinyl-CoA ligase beta chain, a protein related to the tricarboxylic acid cycle.

**Conclusions/Significance:**

Our findings may point to the mechanisms used by highly virulent *P*. *brasiliensis* isolates to withstand host immune reactions and to adapt to transient iron availability as strategies to survive and overcome stress conditions inside the host.

## Introduction

Paracoccidioidomycosis (PCM) is the most frequent endemic systemic mycosis in Latin America with high incidence in Brazil, Argentina, Colombia, and Venezuela [1, 2]. It is caused by the thermally dimorphic species of the genus *Paracoccidioides*. Until recently, *Paracoccidioides* was considered a monotypic taxon typified by *Paracoccidioides brasiliensis* [3]. However, the introduction of molecular phylogenetics shed light on the taxonomy of *Paracoccidioides*, leading to the description of new cryptic entities. To date, four phylogenetic species are recognized inside the *P*. *brasiliensis* complex: S1, PS2, PS3 and PS4 [4, 5]. *P*. *brasiliensis sensu stricto* (*s*. *str*.), formerly known as S1, is the most widely distributed agent of PCM, occurring in Brazil, Argentina, Paraguay, Uruguay, Peru and Venezuela [6]. *Paracoccidioides americana* (formerly known as PS2) occurs in Venezuela and Brazil, in sympatry with *P*. *brasiliensis s*. *str*. [6]. *Paracoccidioides restrepiensis* (PS3) and *P*. *venezuelensis* (PS4) are geographically restricted to Colombia and Venezuela, respectively [6]. Finally, *P*. *lutzii*, an ancient divergent of the *P*. *brasiliensis* complex occurs in Brazil with its epicenter in the Central-West region [7, 8]. A recent speciation event is assumed for species embedded in the *P*. *brasiliensis* complex (especially PS3 and PS4), whereas it seems that *P*. *brasiliensis sensu lato* (*s*.*l*.) and *P*. *lutzii* are reproductively isolated in nature [6].

The ecological niche or exact habitat of these species remains poorly understood [9]. *Paracoccidioides brasiliensis s*. *str*. and *P*. *americana* has been described in armadillos, but not *P*. *lutzii*. The nine-banded armadillo (*Dasypus novemcinctus*) is a natural reservoir of the fungus and animal infection due to *Paracoccidioides* spp. has been observed repeatedly in several endemic areas of Brazil and Colombia [10]. Also, culture-independent surveys based on DNA detection techniques revealed that *P*. *brasiliensis* complex and *P*. *lutzii* are present in the soil [11]. It is accepted that conidia present in nature are inhaled by patients, transformed into budding yeast cells in the lungs, and then, these cells spread to different organs [12]. PCM may manifest itself in a variety of clinical forms, ranging from a benign and localized condition to a more severe and disseminated disease, depending on the extent of the depression of cellular immunity [12–14].

*Paracoccidioides* spp. are able to cause disease symptoms in the murine model. However, different isolates are not homogeneous in their virulence characteristics, a fact that could explain different clinical forms of PCM. The virulence profile of *Paracoccidioides* spp. isolates has been shown to depend upon mycological properties [15], isolate origins [16, 17], species identity [18], genetic patterns [19–21], adhesion process [22–24], activation of immune response [25, 26], culture conditions [27–29], antigenic characteristics [17], and protein levels [17, 30].

Cell-mediated immunity is the predominant host defense mechanism against fungal infections [31]. The role of antibodies in protective immunity during fungal infections can be achieved through many mechanisms, such as neutralization of fungal PAMPS (Pathogen Associated Molecular Patterns), opsonization of fungi and facilitation of their phagocytosis by recognition by Fc receptors present in phagocytes, activation of the classic pathway of the complement system, antibody dependent cell cytotoxicity and direct inhibition of fungal growth.

Though, many breakthrough studies have dissected the role of antibodies in antifungal immunity, as reviewed by Casadevall & Pirofski [32], it is not well characterized how the specific antibodies can mediate this protection [33]. In paracoccidioidomycosis, a robust set of evidences points towards the conclusion that cell-mediated immunity is the main host defense during *P*. *brasiliensis* infection, as stated as early as in 1988 by Castaneda and colleagues [34].

Although the role of specific antibodies as effector molecules of the adaptive immune response is classically known and accepted, their role in providing protective immunity in fungal infections, restricting fungal burden and enhancing their clearance is not a consensus because antibodies are frequently associated with severe PCM. Therefore, it is in dispute whether specific anti-*P*. *brasiliensis* contribute to susceptibility or merely constitute a marker of infection severity or even are protective.

**Fig 1:**
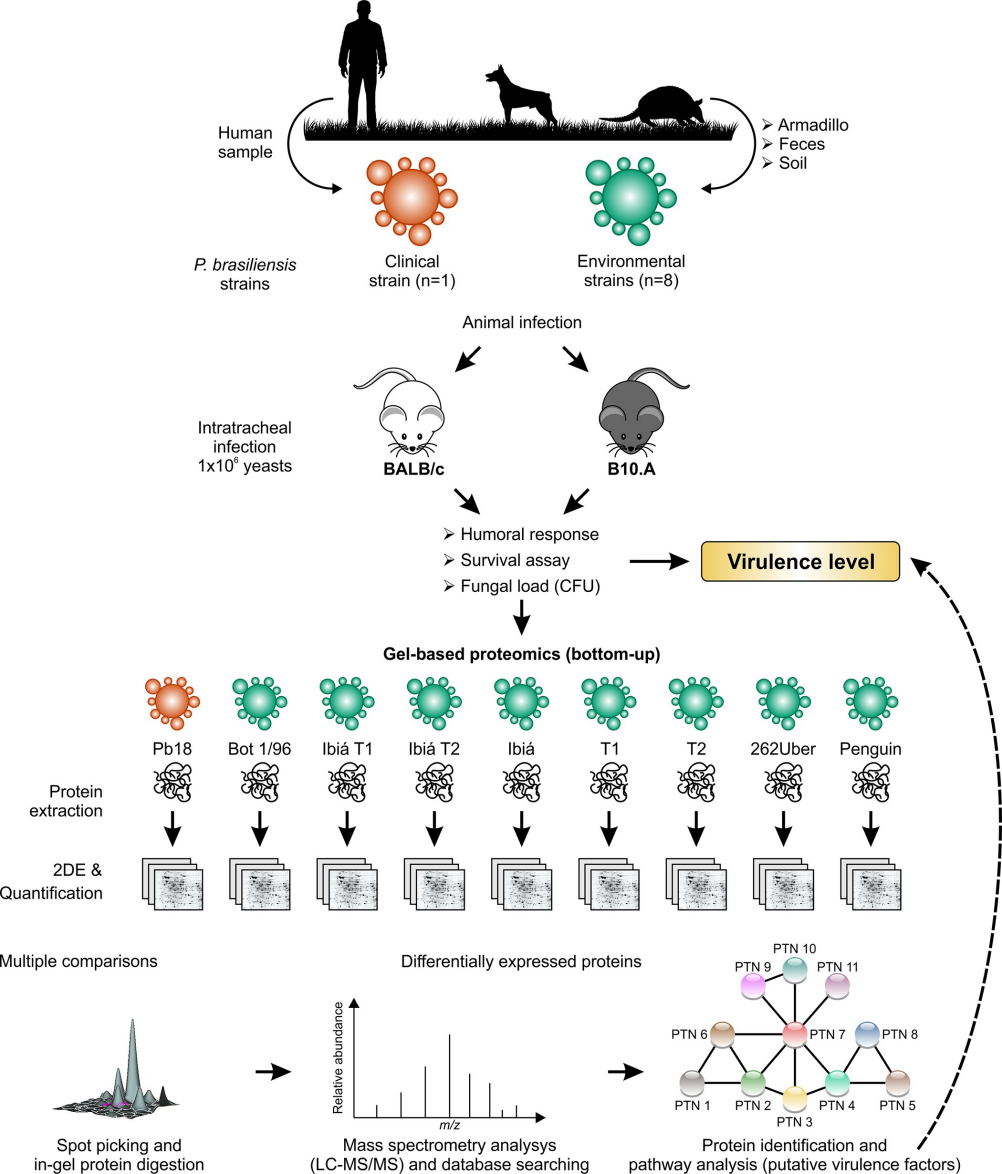
Schematic depicting a two-dimensional (2D) proteomic approach used in this study in an attempt to identify possible virulence factors of *Paracoccidioides* spp. Isolates were selected according to their source (clinical and environmental) and submitted to virulence assays in BALB/c and B10.A mice. Afterwards, the yeasts were recovered, proteins were extracted and then resolved by 2D gel electrophoresis. Proteins were deemed to have differential abundance levels if their spot volumes were changed at least twofold compared to the normalized spot volume, and based on statistical significance. Proteins with differential abundance levels were identified by mass spectrometry analysis. Finally, based on virulence and proteomic assays we classified isolates according to their virulence level.

For host invasion and colonization, *Paracoccidioides* spp. must be able to survive in hostile environments, which requires the presence of various regulatory mechanisms and expression of different virulence factors. These mechanisms allow the fungus to grow at body temperature, adhere to host cells, evade host defenses, and spread to other organs and tissues [23]. The identification of key virulence factors required for disease progression is critical for understanding the biology of *Paracoccidioides* spp. infection. Thus, in the present study, we determined protein levels of *P*. *brasiliensis* complex isolates and correlated them with their virulence characteristics in a murine model. In particular, we evaluated eight *P*. *brasiliensis* complex strains obtained from different environmental and animal sources and compare them to the highly virulent Pb18. The isolates were grouped according to virulence levels, and protein profiles were determined by a two-dimensional (2D) proteomic approach in an attempt to identify possible virulence factors that could correlate with the extent of immunological disturbances observed in the experimental murine PCM model (Fig 1).

## Materials and methods

### Ethical approval

The study was performed in strict accordance with recommendations in the Guide for the Care and Use of Laboratory Animals of the National Institutes of Health and approved by the Institutional Ethics in Research Committee of the Federal University of São Paulo (protocol number 0692/05).

### Fungal isolates

Table 1 shows the origin and location of *P*. *brasiliensis* complex isolates used in this study. These isolates were characterized down to species level by PCR-RFLP of the alpha-tubulin gene as described earlier [35]. Yeast-form samples from all isolates were cultivated in semi-solid Fava Netto's medium [36] at 36°C. The fungi were used at the 7th day in culture, which corresponds to the exponential phase of growth [37]. Attenuation of virulence may occur more rapidly in some *Paracoccidioides* strains than others when subjected to successive *in vitro* subculturing [27, 28] and therefore impact multiple comparisons as proposed here. To prevent any bias among *Paracoccidioides* spp. isolates at the start of *in vitro* culturing, all isolates were passed through BALB/c and then re-isolated prior to challenge experiments.

**Table 1:** Origin and location of *P*. *brasiliensis* complex isolates used in this study.

Isolate	EPM code^1^	Genetic group/ Phylogenetic species^2^	Origin	Location	References
Pinguim	EPM 34	PS3 (*P*. *restrepiensis*)	Animal (Penguin feces)	Uruguay	[38]
Ibiá T1	EPM 101	S1 (*P*. *brasiliensis s*. *str*.)	Animal (Armadillo)	Ibiá (Minas Gerais), Brazil	[39]
Ibiá T2	EPM 102	S1 (*P*. *brasiliensis s*. *str*.)	Animal (Armadillo)	Ibiá (Minas Gerais), Brazil	[39]
Bot 1/96	EPM 11	S1 (*P*. *brasiliensis s*. *str*.)	Animal (Armadillo)	Botucatu (São Paulo), Brazil	[40]
T1	EPM 53	S1 (*P*. *brasiliensis s*. *str*.)	Environment (Soil)	Miranda (Paracotos), Venezuela	[41]
T2	EPM 54	PS3 (*P*. *restrepiensis*)	Environment (Soil)	Miranda (Paracotos), Venezuela	[41]
262Uber	EPM 28	S1 (*P*. *brasiliensis s*. *str*.)	Dog food contaminated with soil	Uberlândia (Minas Gerais), Brazil	[42]
Ibiá	EPM 30	PS3 (*P*. *restrepiensis*)	Environment (Soil)	Ibiá (Minas Gerais), Brazil	[43]
Pb18	EPM 16	S1 (*P*. *brasiliensis s*. *str*.)	Human	São Paulo, Brazil	[44]

^1^Paulista School of Medicine (EPM), Federal University of São Paulo

^2^Molecular characterization based on *TUB1*-RFLP.

### Mice and infection

B10.A and BALB/c isogenic male mice (8 to 12 week-old) were provided by the animal facility of the Federal University of São Paulo, Brazil. Animals were divided into 10 groups of 5 mice each (one group for each *Paracoccidioides* isolate and one negative control group). Animals were housed in temperature-controlled rooms at 23–25°C, five per cage, in standard boxes with *ad libitum* access to food and water. Mice were infected intratracheally (i.t.) with 10^6^ *P*. *brasiliensis* yeast cells/animal. The yeast cells were washed three times in phosphate-buffered saline (PBS), and fungal suspensions were used at a concentration of 1 × 10^6^ cells per 50 μL, adjusted after counting with a hemocytometer. The viability of fungal cells was evaluated using the vital dye Trypan blue as previously described [45] and was always higher than 95%. The control group received 50 μl of PBS only.

### Fungal loads

The severity of the infection was determined by the Colony Forming Unit (CFU) assay using a total of 90 mice. Thirty days after i.t. administration of fungal cells, the animals (n = 5) were euthanized by CO_2_ anesthesia and organs such as the lungs, liver, and spleen were macerated, seeded on Petri dishes containing Brain Heart Infusion agar and incubated at 36°C. The colonies were counted on the 10th day of plating, when the number of colonies was no longer increasing [46].

### Purification of gp43 antigen

Mycelial form samples of *P*. *brasiliensis* isolate B-339 (ATCC 32069; PS3) were cultivated in solid Sabouraud dextrose agar (Difco Laboratories, Detroit, MI, USA) at room temperature. The fungus was converted to the yeast form on modified Sabouraud dextrose agar (Sab-T-A) containing 0.01% thiamine and 0.14% asparagine (Difco Laboratories, Detroit, MI, USA) at 35°C. Exoantigen was produced according to the method of de Camargo *et al*. [47, 48], and gp43 antigen was purified from that exoantigen [49]. Concentrations of purified protein were determined by the Bradford method [50]. Protein fractions were submitted to sodium dodecyl sulfate polyacrylamide gel electrophoresis (SDS-PAGE) [51] and silver stained [52] to confirm the purification. Gp43 antigen was stored at -20°C until use.

### Antibody detection

B10.A and BALB/c mice were infected as described above. Serum samples were obtained by bleeding the tail vein at the 30th day post-infection and stored at -20°C until use. For the indirect enzyme-linked immunosorbent assay (ELISA), polystyrene 96-well microplates (Costar, Corning Inc., Cambridge, MA, USA) were coated with purified gp43 (250 ng/well) diluted in 0.1 M carbonate-bicarbonate buffer, pH 9.6 and incubated at 37°C for 2 h and overnight at 4°C. The plates were washed three times with a 0.05% solution of Tween-20 in PBS (PBS-T), and free sites were blocked with 5% skim milk in PBS-T (200 μL/well) at 37°C for 2 h. After three washes, 100 μL of mouse serum samples diluted at 1:50 in PBS-T (containing 0.25% gelatin—PBS-T-G) were added to the wells in duplicates. The plates were incubated at 37°C for 1 h and washed again. Then, 100 μL of peroxidase conjugated anti-mouse IgG (y-chain specific; 1:1000 dilution in PBS-T-G; Sigma Chemical Co., St Louis, MO, USA) was added to each well. The plates were incubated at 37°C for 1 h, washed, and then, 100 μL of the substrate solution (5 mg of O-phenylenediamine in 10 mL of 0.1 M citrate phosphate buffer, pH 4.5, plus 10 μL of 30% H_2_O_2_) was added to the wells. After color development, the reaction was stopped by the addition of 50 μL of 4 N H_2_SO_4_. The optical density (OD) values were measured at 492 nm using an ELISA microplate reader (Sunrise absorbance reader, Tecan, Mannedorf, Switzerland).

### Protein extraction

*P*. *brasiliensis* yeast cells were grown for 7 days at 36°C in triplicate on Fava-Netto’s medium, and protein extract was obtained as previously described by Rodrigues *et al*. [53]. Briefly, yeast cells were washed in PBS, centrifuged (5,000×g, 5 min, 4°C), frozen in liquid nitrogen, and disrupted by mechanical maceration. Then, 2 mL of buffer extraction medium (20 mM Tris-HCl, pH 8.8, 2 mM CaCl_2_) containing a cocktail of protease and nuclease inhibitors (1:100; GE-healthcare, Uppsala, Sweden) was added. The extract was vortexed, centrifuged (8,000×g, 15 min), and the supernatant was kept at −80°C until use. Protein concentrations were determined by the Bradford method [50].

### Two-dimensional gel electrophoresis

Before two-dimensional gel electrophoresis (2-DE), proteins were concentrated using a 2-D Clean-up Kit (GE Healthcare, Uppsala, Sweden). Next, 300 μg of total protein was diluted in 250 μL of rehydration solution (7 M urea, thiourea 2 M, 2% CHAPS, DeStreak 1,2%, 1% vol/vol isoeletric focusing buffer pH 3–10, applied into 13-cm immobilized pH gradient (IPG 3–10) (GE Healthcare, Uppsala, Sweden), and rehydrated at 20°C for 12 h using an Ettan IPGphor III system (GE Healthcare, Uppsala, Sweden). The rehydrated strips were focused at 20°C as follows: 1 h at 500 V, 1 h at 1000 V, 4 h at 8000 V, 6 h at 8000 V, and 12 h at 1000 V. Focused IPG strips were sequentially incubated for 2×20 min in two equilibration buffer solutions (6 M Urea, 50 mM Tris HCl, pH 6.8, 30% glycerol, 2% SDS), containing 10 mg/mL dithiothreitol and 25 mg/mL iodoacetamide, respectively. The second dimensional separation was performed on 10% polyacrylamide gels (45 mA per gel, 10°C) using a Hoefer SE 600 system (GE Healthcare, Uppsala, Sweden). The gels were stained with Coomassie Brilliant Blue G-250 [54]. The experiments were carried out in triplicate. 2-DE gels were scanned on an Image Scanner III (GE Healthcare, Uppsala, Sweden) and analyzed using Image Master 2D platinum 7.0 software (GE Healthcare, Uppsala, Sweden).

### 2-DE gels image and data analysis

The images of the 2-DE gels were captured by ImageScanner at 300 dots/inch, and spots were quantitatively analyzed using Image Master 2D platinum 7.0 software. After automated matching, manual matching was carried out to correct the mismatched or unmatched spots by adding, splitting and removing spots. To compare spots across gels in each *Paracoccidioides* spp., a match set was obtained with images from all gels, and only well-resolved spots in all three biological replicates were considered reproducible. For the matched protein spots in each 2-DE gel, their volumes were normalized to the total spot volume using the software ImageMaster 2D Platinum 7.0, in order to eliminate the possible variations due to staining. The normalized volume of each protein spot was used as its expression abundance. All values are presented as mean ± S.E.M (standard error of the mean). Proteins were deemed to have differential abundance levels if their spot volumes were changed at least twofold (>2.0-folds) compared to the normalized spot volume, and based on analysis of variance (ANOVA, *P* < 0.05), implemented in the ImageMaster 2D Platinum 7.0.

### Protein digestion and peptide extraction

Digestion was performed according to Pitarch *et al*. [55]. Briefly, the spots were excised from 2-DE gels, destained twice with 50% acetonitrile (ACN) in 25 mM NH_4_HCO_3_, and vacuum-dried. The proteins were then reduced with 10 mM dithioerythritol in 25 mM NH_4_HCO_3_ for 30 min at 56°C and alkylated with 55 mM iodoacetamide in 25 mM NH_4_HCO_3_ for 20 min in the dark. Afterwards, gel pieces were washed with 25 mM NH_4_HCO_3_ and ACN, and dried under vacuum. All gel pieces were incubated with 12.5 ng/mL sequencing grade trypsin (Promega, Madison, WI, USA) in 25 mM NH_4_HCO_3_ overnight at 37°C. Peptides were then extracted from the gel pieces with 50% ACN, 1% trifluoroacetic acid solution in 25 mM NH_4_HCO_3_, and finally with 100% ACN. The combined extracts were dried in a SpeedVac concentrator (Thermo Fisher Scientific, Waltham, MA, USA). Samples were then subjected to mass spectrometry analysis.

### Liquid chromatography-tandem mass spectrometry (LC-MS/MS) analysis

A 4.5-μL aliquot of digested proteins was injected into a C18 1.7 μm BEH 130 (100 μm × 100 mm) RP-ULC analytic column (nanoAcquity UPLC, Waters Corporation, Manchester, UK) coupled with nano-electrospray tandem mass spectrometry system on a Q-Tof Ultima API mass spectrometer (MicroMass/Waters, Corporation, Manchester, UK) at a flow rate of 600 nL/min. A trapping Symmetry C18 column (180 μm × 20 mm) was used for sample desalting at a flow rate of 20 μL/min for 1 min. The gradient was 0–50% ACN (acetonitrile) in 0.1% formic acid over 45 min. The instrument was operated in the MS positive mode, data continuum acquisition from m/z 100 to 2,000 Da at a scan rate of 1 s and inter-scan delay of 0.1 s.

### Database search

Database searches for the identification of peptides from LC MS-MS experiments were done with Mascot Distiller v.2.3.2.0, 2009 (Matrix Science, Boston, MA) using carbamidomethyl-cys as fixed modification (monoisotopic mass 57.02015 Da), oxidation (HW) and oxidation (M) as variable modification (monoisotopic mass 15.0215 15.9949), and 0.1 Da MS and MS/MS fragment tolerances. After the analysis, the data from each spot were exported in a text file format. Sequence database search was carried out with MASCOT search engine (Matrix Science Ltd., London, UK). The results were compared to known sequences from *Paracoccidioides* database (http://www.broadinstitute.org/) [56, 57]. The default significance threshold was *P* < 0.05.

### Statistical analysis

CFU assay and ELISA results were assessed statistically by using the two-way ANOVA followed by pairwise comparisons by the *post hoc* Tukey’s test. Differences were considered statistically significant if corresponding *P*-values were below 0.05.

## Results

### Experimental paracoccidioidomycosis

**Fig 2:**
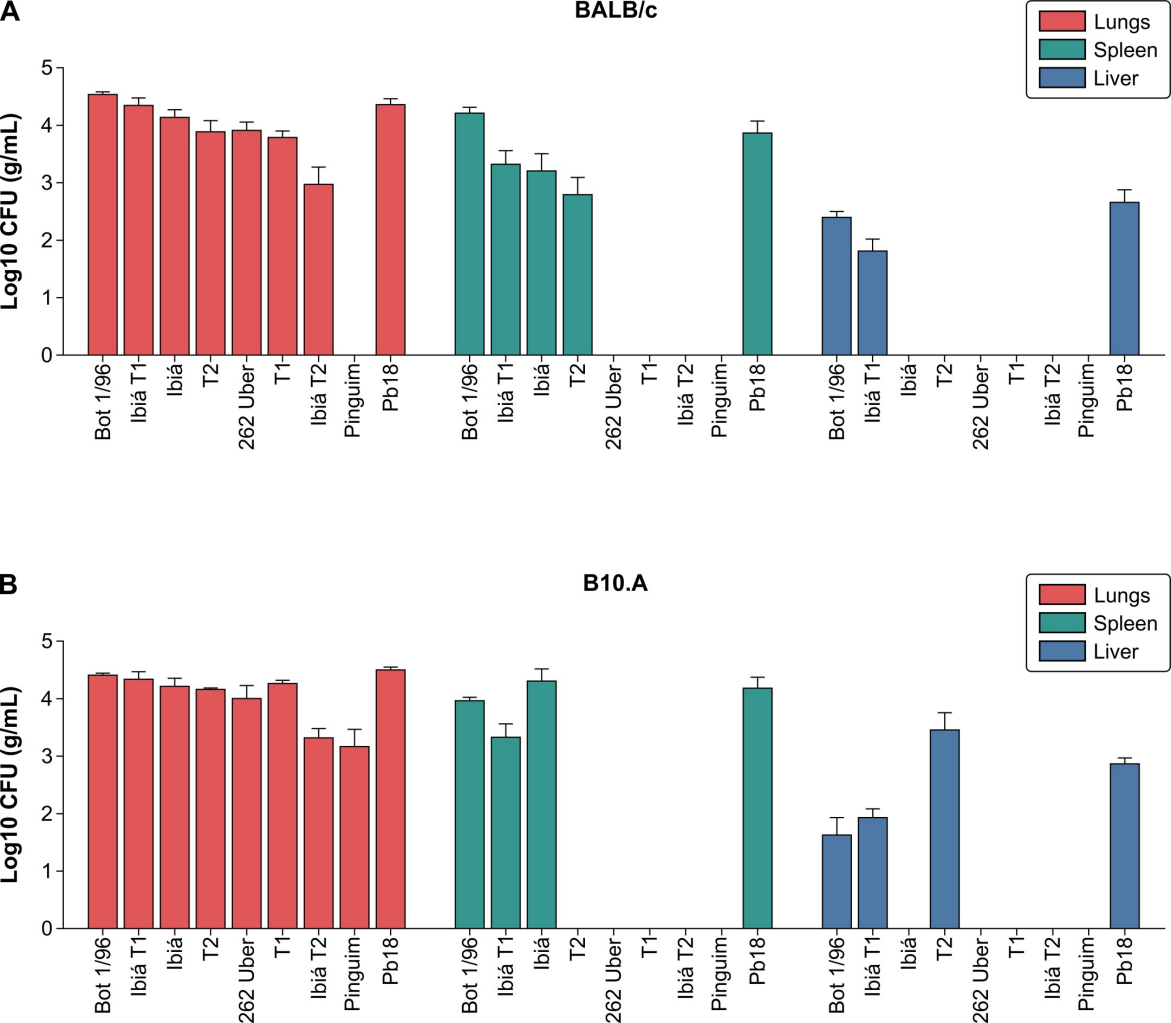
Fungal loads in different mouse tissues. Colony-forming units (CFUs) were obtained from samples of the spleen, liver, and lungs of BALB/c (A) and B10.A (B) mice infected intratracheally with 1×10^6^ *P*. *brasiliensis* yeast cells. Mice were euthanized at 30 days after inoculation. Data are representative of two independent experiments and values are expressed in mean ± SD. Horizontal bars indicate statistical significance of differences between numbers of CFUs obtained from isolate samples and those of virulent control Pb18: *P < 0.05, Tukey’s test.

The fungal tissue burden in the liver, spleen, and lungs for the BALB/c (Fig 2A) and B10.A mice (Fig 2B) inoculated with one of the nine *P*. *brasiliensis* complex isolates studied is shown in Fig 2. All isolates except Pinguim isolate were able to colonize the lungs, the target organ of inoculation in both mouse lines. Bot 1/96 and Ibiá T1 isolates that colonized mouse spleen and liver caused disseminated disease and, therefore, were considered highly virulent and comparable to control Pb18 *P*. *brasiliensis* strain. Ibiá and T2 isolates colonized only the spleen besides the lungs and induced a less severe form of the disease. These isolates were therefore classified as moderately virulent. The remaining isolates (262Uber, T1, and Ibiá T2) caused limited disease manifestations and affected only the lungs, being unable to disseminate to other organs. These isolates were considered to possess low virulence. It was not possible to recover the fungi from any examined organs of BALB/c mice infected with Pinguim isolate. At the same time, viable fungi could be recovered from the lungs of infected B10.A mice (Fig 2B). Similar patterns of fungal dissemination were observed in both mouse strains, except for those of T2 and Pinguim isolates. T2 isolate disseminated to the spleen in BALB/c mice and to the liver in B10.A mice. During the thirty days post inoculation with *P*. *brasiliensis* complex isolates, no deaths were observed in the two mouse strains.

**Fig 3:**
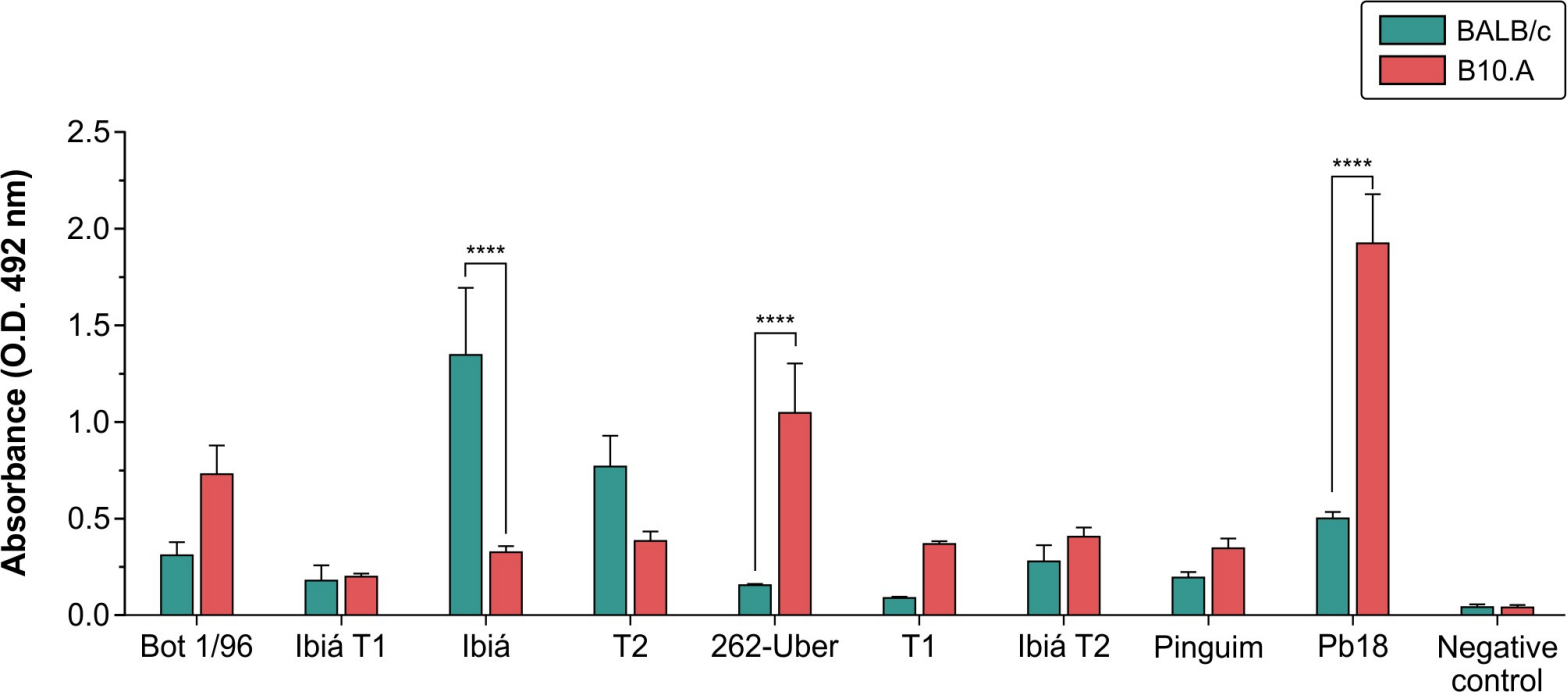
Anti-gp43 antibody detection. The levels of anti-gp43 antibody were determined in sera from B10.A and BALB/c mice by ELISA. Groups of five mice each were inoculated intratracheally with 10^6^ *P*. *brasiliensis* yeast cells. Serum samples were obtained by tail vein bleeding at the 30^th^ day post-infection. Serum samples from non-infected mice were used as negative control. Data are representative of two independent experiments and values are expressed in mean ± SD. Statistical significance of differences is indicated as follows: ****P <0.0001, Tukey’s test.

We then evaluated the humoral immune response of BALB/c and B10.A mice infected with *Paracoccidioides* isolates as described above (Fig 2). Fig 3 shows that all *P*. *brasiliensis* complex isolates induced the production of anti-gp43 antibodies. However, the isolates elicited distinct serological responses during infection. We observed that BALB/c mice infected with moderately virulent Ibiá and T2 isolates had higher antibody titers than highly virulent control Pb18 *P*. *brasiliensis* strain. Statistical analysis revealed that there was no correlation between the degree of *P*. *brasiliensis* complex isolate virulence (Fig 2) and antibody production in mice. The data on the virulence and dissemination of *P*. *brasiliensis* complex isolates are summarized in Table 2.

**Table 2:** Virulence characteristics of *P*. *brasiliensis* complex isolates based on murine model of infection.

*P*. *brasiliensis* isolate	Dissemination in BALB/c mice	Dissemination in B10.A mice	Mortality	Virulence level
**Bot 1/96**	Spleen and liver	Spleen and liver	No	High
**Ibiá T1**	Spleen and liver	Spleen and liver	No	High
**Ibiá**	Spleen	Spleen	No	Medium
**T2**	Spleen	Liver	No	Medium
**262 Uber**	None	None	No	Low
**T1**	None	None	No	Low
**Ibiá T2**	None	None	No	Low
**Pinguim**	None	None	No	Low
**Pb18**	Spleen and liver	Spleen and liver	No	High

### Comparative proteomic analysis of *P*. *brasiliensis* complex isolates

**Fig 4:**
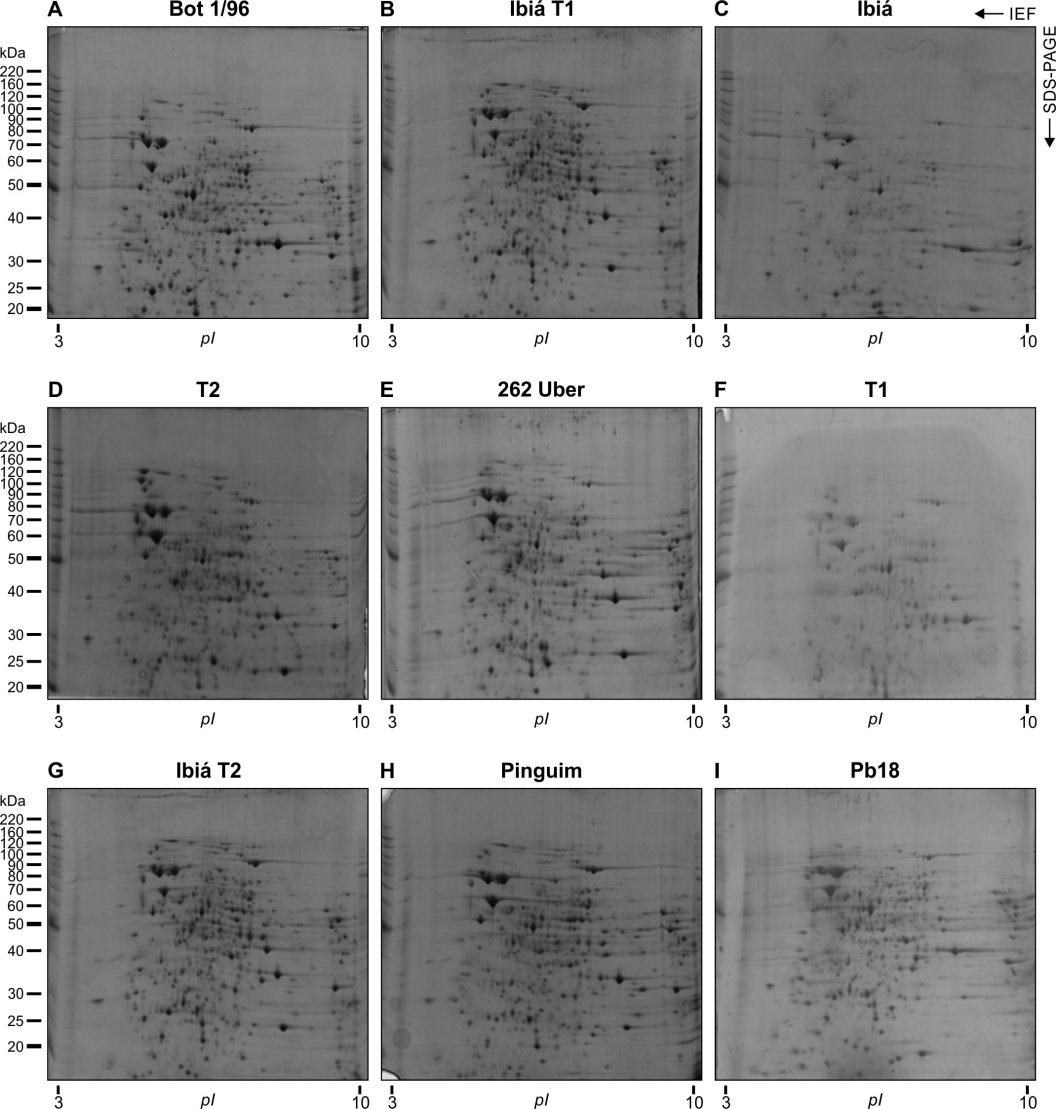
Proteomic maps of *P*. *brasiliensis* complex isolates. Cell extracts (originated from three biological replicates) were subjected to 2D electrophoresis on 13-cm immobilized pH gradient strips in the range of *pI* values from 3 to 10 (GE Healthcare, USA), and the proteins were developed by Coomassie blue staining. A representative 2D gel is shown for each *Paracoccidioides* isolate; a: Bot 1/96, b: Ibiá T1, c: Ibiá, d: T2, e: 262 Uber, f: T1, g: Ibiá T2, h: Pinguim, i: Pb18. Molecular masses of standard proteins are given on the left side of the gel (BenchMark Protein Ladder, Invitrogen). Further information about protein levels using 1D gel can be found in the S1 Fig.

Comparative analysis of proteins up- or down-regulated in *P*. *brasiliensis* complex isolates with highly, moderate, and low virulence was performed in order to determine the proteins whose levels correlates with the extent of virulence. For this purpose, protein extracts of the nine isolates (Table 1) were fractionated by 2D electrophoresis in triplicate (pH 3–10). Representative images of proteome maps from *P*. *brasiliensis* complex isolates are shown in Fig 4.

**Fig 5:**
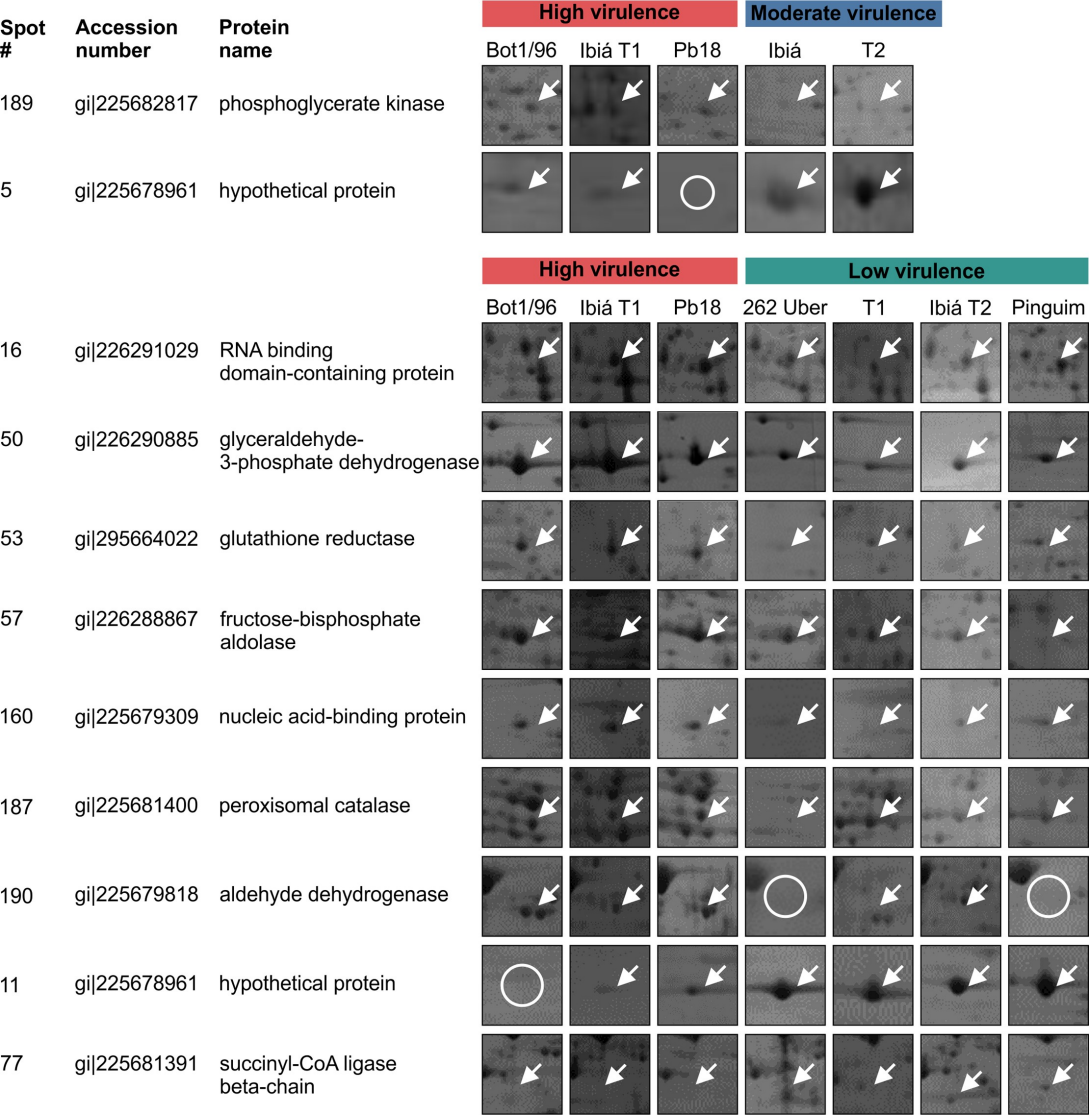
Identification of proteins by LC-MS/MS. Proteins corresponding to the spots of interest in 2D electrophoresis gels were identified by LC-MS/MS. An arrow indicates the presence of the protein, whereas an open circle indicates the absence of the protein. A: Proteins with differential abundance levels between highly and moderately virulent isolates. B: Proteins with differential abundance levels between highly and slightly virulent isolates. The data presented represent the results of three biological replicates.

Gel image analyses were conducted using protein profiles of highly virulent isolates (Bot 1/96, Ibiá, and Pb18) as references that were compared to profiles of isolates with moderate (Ibiá and T2) and low (262-Uber, T1, Ibiá T2, and Pinguim) virulence. Qualitative analyses were performed considering the presence or absence of particular protein spots, depending on biological properties, i.e., by noting whether the corresponding isolate had high, moderate, or low virulence. Quantitative comparisons were carried out by focusing on spots that exhibited two-fold variations in intensity. By comparing highly and moderately virulent isolates, we revealed 25 spots corresponding to the proteins that were strongly expressed by highly virulent Bot 1/96, Ibiá, and Pb18 isolates, as well as five protein spots that were denser in moderately virulent Ibiá and T2 isolates (S1 Table). The comparative analysis of highly virulent Bot 1/96, Ibiá, and Pb18 isolates with 262 Uber, T1, and Ibiá T2 strains with low virulence showed that 29 proteins were up-regulated in the former isolates and five proteins had higher density in isolates of low virulence (S2 Table). Protein spots up-regulated in both biological conditions were analyzed by mass spectrometry (Table 3). The identified proteins were located in the proteomic maps of *P*. *brasiliensis* complex isolates (Table 1) and are shown in Fig 5.

**Table 3:** Identification of protein spots from 2D electrophoresis gels from *P*. *brasiliensis* complex isolates by LC-MS/MS.

Experimental condition	Isolate virulence	Spot number^1^	Accession number^2^	Protein name	Experimental MM^3^ (kDa)/pI^4^	Theoretical MM^3^ (kDa)/pI^4^	% seq. cov.^5^	Protein score
**Highly versus moderately virulent isolates**	High	189	gi|225682817	phosphoglycerate kinase	43.37/5.24	45.13/6.28	10%	153
	Moderate	5	gi|225678961	predicted protein	23.37/8.21	24.61/8.49	17%	203
**Highly versus slightly virulent isolates**	High	16	gi|226291029	RNA binding domain-containing protein	25.5/5.95	30.53/9.20	24%	395
		50	gi|226290885	glyceraldehyde-3-phosphate dehydrogenase	33.71/8.07	36.57/7.12	49%	687
		53	gi|295664022	glutathione reductase	35.09/4.98	51.95/6.74	10%	302
		57	gi|226288867	fructose-bisphosphate aldolase	37.5/6.90	39.74/6.28	26%	371
		160	gi|225679309	nucleic acid-binding protein	43.27/4.49	10.88/5.77	2%	44
		187	gi|225681400	peroxisomal catalase	54.70/7.30	57.65/6.42	4%	102
		190	gi|225679818	aldehyde dehydrogenase	55.00/5.37	54.24/5.30	2%	38
	Low	11	gi|225678961	predicted protein	24.00/8.20	24.61/8.49	12%	146
		77	gi|225681391	succinyl-CoA ligase beta-chain	46.25/5.11	48.56/5.61	15%	310

^1^Spot numbers refers to S1 and S2 Tables

^2^GenBank general information identifier

^3^Molecular mass

^4^Isoeletric point

^5^sequencing coverage (%)

## Discussion

In this study, we used a proteomic approach to identify proteins differentially expressed by *P*. *brasiliensis* complex isolates that exhibited different levels of virulence. Our main objective was to correlate biological roles of these proteins to respective virulence level. A total of eight up-regulated proteins were successfully identified by LC-MS/MS in highly virulent isolates. One up-regulated protein was identified in a moderately virulent isolate, and two—in isolates of low virulence. Unfortunately, other proteins with differential abundance levels could not be identified, probably because their amounts were too low to produce a good spectrum, or because the confidence levels of the database search were insufficient to yield unambiguous results.

According to CFU assays, the isolates were classified as those having high (Bot 1/96, Ibiá T1 and Pb 18), moderate (Ibiá and T2), or low (262Uber, T1, Ibiá T2, and Pinguim) virulence. A very similar pattern of fungal dissemination in BALB/c and B10.A mice was observed for the majority of isolates except for that of Pinguim isolate, which was unable to colonize any organs of BALB/c mice, but could be recovered from the lungs of B10.A mice. In relevance to this result, it has been shown previously that B10.A mice were highly susceptible, whereas BALB/C mice were moderately susceptible to *P*. *brasiliensis* Pb18 infection by intraperitoneal route [58]. In addition, the isolate Pinguim, which was previously considered a new *Paracoccidioides* species, *P*. *antarcticus* [MB#492297], was isolated from *Pygoscelis adeliae* faeces (a penguin found along the entire Antarctic coast and at some of its nearby islands), is capable of producing experimental orchids in guinea pigs [59]. *In vitro* cultivation at 36°C reveals marked morphological variation in the yeast phase [59], but antigenic preparations displayed total immunological identity with classical *P*. *brasiliensis* strains, mainly regarding the specific gp43 component [60], as detected here using ELISA. From a phylogenetically point of view, Pinguim is classified as *P*. *brasiliensis* S1, a paraphyletic group, and shares genetic similarity with other atypical isolates (e.g. JT-2, 1430) [61]. The diversity of morphology, genetic and proteomic characteristics may support the variety of responses during interaction with different hosts as observed here for the BALB/C and B10.A mice.

In our experiments, we evaluated both the ability of *P*. *brasiliensis* complex isolates to induce immune response in mice and possible correlation between the level of isolate virulence and antibody titer. We observed that all isolates, including Pinguim isolate, were able to induce the production of anti-gp43 antibodies in both BALB/c and B10.A strains of infected mice, indicating that the infection occurs even if the corresponding isolate cannot be readily recovered from mouse organs.

Furthermore, the statistical analysis revealed that there was no correlation between isolate virulence and induced antibody response. Our results showed that although the isolates had varying immunogenicity levels, these differences were not sufficient to explain either the severity of infection or tissue tropism. In a fact, our results are in disagreement with several influential publications on human PCM, which posited that severe forms of the disease are associated with the highest antibody titers [12, 62]. Discrepant results regarding the relationship between antibody response and severity of clinical forms of PCM have been also documented by other researchers [63–65]. Singer-Vermes *et al*. [66] examined six *P*. *brasiliensis s*.*l*. isolates obtained from samples of patients that presented with distinct and well-defined clinical forms of PCM and compared their virulence, tissue tropism, and humoral immune response in susceptible B10.A mice. They found that in general, pathogenicity and immunogenicity parameters in humans and in susceptible mice were not analogous. The authors argued that in human body, unlike in experimental laboratory conditions, PCM has a very slow evolution. In addition, factors related to the host, such as genetic pattern, sex, age, nutritional and immunological status may be of greater relevance to the evolution and outcome of PCM.

The reports showing that humoral immune responses play an important role in conferring protection against PCM are still scarce. In experimental PCM, B lymphocytes-knockout mice were shown to be more susceptible to *P*. *brasiliensis* infection than their wild-type controls, presenting higher mortality rate and numbers of viable *P*. *brasiliensis* in the lungs. The granulomas of the knock-out mice were larger than those of the control mice, and as the size and organization of *P*. *brasiliensis* granulomas reflect the control or not of the infection [67], the results point towards a protective effect of B lymphocytes. The absence of B cells leads to increased levels of IL-10, confirming experimental data that links this observation with more severe disease. Therefore, this data suggests that in experimental PCM B lymphocytes are paramount to effectively control both *P*. *brasiliensis* growth and the organization of the granulomatous lesions [68].

A report by Montagnoli and colleagues revealed that antibodies have a critical role in the generation of memory antifungal immunity [69]. Also working with mice deficient in B lymphocytes, Montagnoli *et al*. showed that although passive administration of antibodies increased the fungal clearance, the innate and Th1-mediated resistance to the primary and secondary infections were both heightened in mu MT mice with candidiasis and aspergillosis. However, although capable of efficiently restricting the fungal growth, mu MT mice did not survive the re-infection with *Candida albicans*, and this was concurrent with the failure to generate IL-10-producing dendritic cells and regulatory CD4(+)CD25(+) T cells. Antifungal opsonizing antibodies restored IL-10 production by dendritic cells from mu MT mice, a finding suggesting that the availability of opsonizing antibodies may condition the nature of the dendritic cell interaction with fungi, possibly impacting on the development of long-lasting antifungal immunity [69].

IgG2a and IgG2b monoclonal antibodies against the major diagnostic antigen of *Paracoccidioides brasiliensis*, gp43 were shown to reduce fungal burden and was associated with the enhanced phagocytosis of *P*. *brasiliensis* by macrophages leading to increased nitric oxide production. The monoclonal antibody against the major diagnostic antigen of *P*. *brasiliensis* mediates immune protection in infected BALB/c mice challenged intratracheally with the fungus [70].

Although humoral immunity might not have a major role in conferring protection against fungal infections in human, passive administration of specific protective antibodies proved to be beneficial in drug resistance cases, to reduce the dosage and associated toxic symptoms of antifungal drugs.

It was experimentally demonstrated that antibodies produced against gp70, a circulating antigen detected during PCM, prevented the establishment of the disease in mice [71]. Also, the adaptive transference of WT immune or non-immune serum to B-lymphocyte knock-out mice is associated with better clinical features, including diminished infiltration of inflammatory cells and formation of organized granuloma. The authors conclude that B cells are effectively involved in the control of *P*. *brasiliensis* growth and participate in the organization of the granulomatous lesion observed in the lungs from Pb18-infected mice [68].

Our proteomic analysis indicates that highly virulent isolates probably expressed a higher amount of phosphoglycerate kinase than isolates of low virulence. Furthermore, highly virulent isolates had higher levels of RNA binding domain-containing protein (RBP), glyceraldehyde-3-phosphate dehydrogenase, glutathione reductase, fructose-bisphosphate aldolase, nucleic acid-binding protein, peroxisomal catalase, and aldehyde dehydrogenase (ALDH) than isolates of low virulence. Judging from the proteins above, there is a connection between pathogenicity, metabolism, and redox homeostasis. Energy metabolism is largely from glycolysis, a metabolic pathway that is fundamental to the assimilation of carbon for either respiration or fermentation, and therefore is critical for the growth of *Paracoccidioides* and other fungal pathogens [72, 73]. Our results support the up-regulation of genes involved in gluconeogenesis in highly virulent *Paracoccidioides*, as the protein levels related to carbohydrate metabolism increase. Interestingly, glucose-6-phosphate dehydrogenase, the first enzyme of the pentose phosphate cycle, has the interesting property of reducing NADP+ to NADPH(H)+ and thus is the key enzyme that provides the reducing power of the cell [74]. It has been demonstrated that HeLa cells expressing high levels of glucose-6-phosphate dehydrogenase display an increased level of reduced glutathione and show oxidoresistance [74–76]. In addition, aldehyde dehydrogenase may consolidate intracellular redox homeostasis in *Paracoccidioides* by detoxifying stress-generated aldehydes, an important feature to survive within the human host [73].

It has been shown that proteins may have multiple independent functions. This phenomenon occurs in both eukaryotes and prokaryotes, including the representatives of *Paracoccidioides* genus [77]. In our experiments, we found three glycolytic enzymes whose expression varied with *Paracoccidioides* virulence: phosphoglycerate kinase, glyceraldehyde-3-phosphate dehydrogenase, and fructose-bisphosphate aldolase. Despite their role in carbohydrate metabolism, it has been demonstrated that the relevance of these proteins for PCM pathogenesis stems from their role in adhesion [78, 79], oxidative stress [73], synthesis of extracellular vesicles and cell wall [80, 81], and as immunogenicity [82, 83]. Those reports corroborate our findings, because the above mentioned enzymes were overexpressed in isolates that caused severe and disseminated disease in mice. Thus, the enzymes up-regulated in highly virulent isolates were probably important for shaping virulent phenotype of these strains.

During the infection with *P*. *brasiliensis*, macrophages and neutrophils constitute one of the primary defense mechanisms. These cells generate reactive oxygen and reactive nitrogen species that can damage amino acids, lipids, DNA, and ultimately lead to cell death [84]. To overcome this defense system, intracellular pathogens must have adaptive mechanisms to survive in this hostile environment. In our experiments, we revealed two molecules with protective antioxidant activity, glutathione reductase and peroxisomal catalase, which were up-regulated in highly virulent isolates. Glutathione reductase has been implicated in the virulence of *Cryptococcus neoformans* [85] and *Candida albicans* [86]. In *Paracoccidioides* spp., the importance of glutathione reductase during infection has not been studied in detail. However, this protein has been shown to be up-regulated in mycelial secretome [87] and expressed with differential abundance levels in *Paracoccidioides* species [87, 88]. Peroxisomal catalase has been identified as a typical monofunctional enzyme highly expressed at the yeast phase [82, 89] and up-regulated when the fungus is phagocytosed by macrophages [90, 91]. The exposure of *Paracoccidioides* yeast cells to hydrogen peroxide induced overexpression of peroxisomal catalase [73, 89, 92]. In our study, up-regulation of these proteins in highly virulent isolates may be related to the mechanism by which *P*. *brasiliensis* evades immune system as a strategy for its survival within infected host cells and dissemination to other organs. In contrast, lower expression of these enzymes in isolates with low virulence seems to correspond to their limited ability to cause fungal infection at the inoculation site, the lungs.

Representatives of the ALDH protein superfamily are expressed by species of all three taxonomic domains and are involved in a variety of biological processes, including metabolism of toxic aldehydes and maintenance of the cellular homeostasis [93]. It has been shown that exposure of organisms to stress conditions leads to the increase in ALDH expression [94]. In *Paracoccidioides* genus, expression ALDH has not been extensively studied. However, de Arruda Grossklaus *et al*. [73] demonstrated that yeast cells treated with hydrogen peroxide had higher expression of ALDH than non-treated cells. Recently, Chaves *et al*. [79] demonstrated that ALDH binds to plasminogen, a fact that indicates a potential role of ALDH in the pathogen-host interaction. In our study, the up-regulation of ALDH in highly virulent isolates could be an indication of the involvement of this enzyme in the protection of the fungus from general stress generated by host defense mechanisms.

In the present study, we also detected an increased expression of the RBP and nucleic acid binding protein in highly virulent isolates. RBPs that regulate gene expression at all levels, are numerous and widely distributed in nature. They are key modulators of gene expression and are involved in cell differentiation, cellular response to environmental changes, and cell death [95]. The relationship between RBPs and post-transcriptional regulation/ stress response was observed in several organisms, such as viruses [96], bacteria [97], protozoa [95], plants [98], and mammals [99]. RBPs have been implicated in the regulation of stress response in pathogenic fungi such as *Aspergillus fumigatus* [100], *Cryptococcus neoformans* [101], and *C*. *albicans* [102–104]. The mechanism by which RBP contributes to the virulence of *Paracoccidioides* is unclear. However, Parente *et al*. [105] showed that *Paracoccidioides* yeast cells exposed to nitrosative stress overexpressed the posttranscriptional regulator mRNA binding protein. Our present study suggests that RBP is important to *Paracoccidioides* resistance to host defense system, because its presence appears to enhance *Paracoccidioides* virulence *in vivo*. Moreover, during the infection, *Paracoccidioides* species become exposed to a very hostile environment, and the adaptation processes require global reorganization of gene expression, a fact that could explain RBP overexpression in highly virulent isolates.

Iron is essential for supporting infectiveness of many microorganisms, including *P*. *brasiliensis* [106], due to its role in electron transfer and acid-base reactions, and because it acts as cofactor in a variety of biological processes [107]. For these reasons, the host needs to maintain a balance in iron bioavailability to ensure sufficient levels for own cellular metabolism and, at the same time, to limit the availability of iron to pathogens, as a defense measure [108]. In this regard, *Paracoccidioides* spp. developed a mechanism to obtain iron from high-affinity iron-binding proteins, such as hemoglobin [109]. In addition to its iron uptake system, *P*. *brasiliensis* is able to alter its metabolism according to iron availability, e.g., by inducing the expression of glycolytic pathway proteins at high iron concentrations or by repressing the expression of tricarboxylic acid cycle (TCA) proteins under conditions of limited iron availability, as TCA reactions are mediated by enzymes containing Fe/S [110]. In our experiments, we found that succinyl-CoA ligase beta chain, a protein related to TCA, was down-regulated in highly virulent isolates whereas phosphoglycerate kinase, glyceraldehyde-3-phosphate dehydrogenase, and fructose-bisphosphate aldolase were overexpressed by highly virulent isolates. These findings may indicate a mechanism used by virulent *P*. *brasiliensis* complex isolates to adapt to transient iron availability as a strategy to survive and overcome stress conditions inside the host. Interestingly, glyceraldehyde-3-phosphate dehydrogenase and fructose-bisphosphate aldolase, which were overexpressed by highly virulent isolates in our experiments, were also found in *Paracoccidioides* extracellular vesicles preparations of Pb18 isolate [111]. Such elegant vesicular transport may deliver substances across *Paracoccidioides* cell wall, possibly modulating the host’s immune response and supporting the high virulence phenotype observed in our murine models.

Another potential virulence factor detected in our analysis was a hypothetical protein (accession number: gi|225678961) with a theoretical molecular mass of 24.61 kDa and *pI* of 8.49 that was overexpressed in isolates of moderate and low virulence when compared to its level in highly virulent isolates. In fact, Desjardins *et al*. [57] reported that nearly 60% of *Paracoccidioides* genes were annotated as those encoding hypothetical proteins with unknown cellular functions, but for which no evidence of *in vivo* expression exists [112]. To the best of our knowledge, this is the first report describing the role of such a protein in *Paracoccidioides*-induced mouse infection. Thus, further studies will be needed to characterize this protein and understand its role in PCM infection.

In summary, the data suggest that highly virulent *P*. *brasiliensis* complex isolates that caused disseminated disease in a murine model of PCM expressed high levels of common proteins, such as phosphoglycerate kinase, RNA binding protein, glyceraldehyde-3-phosphate dehydrogenase, glutathione reductase, fructose-bisphosphate aldolase, nucleic acid-binding protein, peroxisomal catalase, and aldehyde dehydrogenase. These proteins were probably critical to the ability of *P*. *brasiliensis* to colonize the host, to survive in its hostile environment, and to escape host immune system, because they appear to be more abundant in highly virulent isolates than in isolates with low virulence. Although our data are not sufficiently complete to create an integral model of *P*. *brasiliensis* pathogenicity, however, they provide important clues towards understanding how fungi adapt to host immune response. In light of our present observations, these proteins levels with differential abundance levels need to be validated using more isolates from human cases of PCM as well as reverse genetic function analysis [113]. The roles of these proteins in different clinical forms of PCM should be studied in order to determine either their potential to be used as biomarkers or targets of therapeutic treatment.

## Supporting information

S1 Table Comparative analysis of proteomes of highly and moderately virulent *P. brasiliensis* complex isolates. Quantitative analysis of proteins with differential expression in highly virulent isolates (Bot 1/96, Ibiá, Pb18) and moderately virulent isolates (Ibiá and T2) is shown. Positive and negative values indicate higher or lower expression of the protein, respectively. Positive (+) and negative (−) symbols mean the presence or absence of the spot, respectively. Proteins were deemed to have differential abundance levels if their spot volumes were changed at least twofold compared to the normalized spot volume.
(PDF)

S2 Table Comparative analysis of proteomes of *P. brasiliensis* complex isolates with high and low virulence. Quantitative analysis of proteins with differential expression in highly virulent isolates (Bot 1/96, Ibiá, Pb18) and isolates of low virulence (262Uber, T1 and Ibiá T2). Positive and negative values indicate higher or lower expression of the protein, respectively. Positive (+) and negative (−) symbols mean the presence or absence of the spot, respectively. Proteins were deemed to have differential abundance levels if their spot volumes were changed at least twofold compared to the normalized spot volume.
(PDF)

S1 Fig Protein profile from *Paracoccidioides brasiliensis* strains and densitometry measurements along the region of interest (ROI; arrow). **(A)** *Paracoccidioides brasiliensis* yeast cells were grown for 7 days at 36°C in triplicate on Fava-Netto’s medium, and protein extract was obtained as previously described [1]. Protein concentrations were determined by the Bradford method [2] and 5 μg of each extract were subjected to 1D SDS-PAGE (10%). The molecular masses (in kDa) of standard proteins are given to the left of the gel (BenchMarkTM Protein Ladder, Invitrogen). From left to right: Bot 1/96, Ibiá T1, Ibiá, T2, 262 Uber, T1, Pinguim, Ibiá T2 and Pb18. **(B)** The completed electrophoresis gel was imaged on an Image Scanner III (GE Healthcare, Uppsala, Sweden) and the comparison was carried out by densitometry measurements of scanned image (8-bit image) along the ROI (arrow) using a 256 grey level scale to determine the average gray value using Adobe Photoshop CC. This region was chosen because it did not present proteins with different abundance levels in the comparative analyzes (2D-GE). **(C)** Lowest and highest gray values were used to set the ratio between each of the extreme values and the ROI revealing minimum variation across the different samples.
(PDF)
