# Supplementary material for: Proteomic analysis of Paracoccidioides brasiliensis complex isolates: Correlation of the levels of differentially expressed proteins with in vivo virulence
Source: PLoS One. 2019 Jul 2;14(7):e0218013. doi: 10.1371/journal.pone.0218013 (PMC6605636; doi:10.1371/journal.pone.0218013)
Supplement: S1 Table — Quantitative analysis of proteins with differential expression in highly virulent isolates (Bot 1/96, Ibiá, Pb18) and moderately virulent isolates (Ibiá and T2) is shown. Positive and negative values indicate higher or lower expression of the protein, respectively. Positive (+) and negative (−) symbols mean the presence or absence of the spot, respectively. Proteins were deemed to have differential abundance levels if their spot volumes were changed at least twofold compared to the normalized spot volume. (PDF) [file pone.0218013.s001.pdf]

S1 Table. Comparative analysis of proteomes of highly and moderately virulent *P. brasiliensis* complex isolates. Quantitative analysis of proteins with differential expression in highly virulent isolates (Bot 1/96, Ibiá, Pb18) and moderately virulent isolates (Ibiá and T2) is shown. Positive and negative values indicate higher or lower expression of the protein, respectively. Positive (+) and negative (−) symbols mean the presence or absence of the spot, respectively. Proteins were deemed to have differential abundance levels if their spot volumes were changed at least twofold compared to the normalized spot volume.

Spot number	pI	MM	Relative abundance (fold change)		ANOVA
			Highly virulent isolates	Moderately virulent isolates	
5	8,21	23,3	-3,62	+3,62	2,74E-06
6	7,13	24,6	-2,16	+2,16	5,89E-03
28	8,88	34,6	-2,82	+2,82	4,40E-02
32	7,08	37,4	-2,21	+2,21	8,67E-03
41	6,25	46,1	+2,82	-2,82	4,90E-02
45	7,39	50,6	+2,19	-2,19	3,08E-02
56	5,1	59,5	-2,97	+2,97	4,18E-02
84	6,63	28,9	+2,92	-2,92	3,79E-02
90	4,98	34,9	+2,08	-2,08	2,21E-02
123	6,54	60,7	+2,021	-2,02	1,95E-02
139	4,93	25	+2,19	-2,19	1,32E-02
157	7,32	26,75	+2,22	-2,22	7,90E-03
164	7,75	29,25	+2,37	-2,37	1,95E-04
170	7,24	37,5	+2,08	-2,08	1,57E-02
189	5,24	43,37	+2,02	-2,02	2,33E-02
190	5,56	37,87	+2,35	-2,35	7,97E-03
224	6,89	55,25	+2,39	-2,39	6,83E-03
244	7,51	67,25	+3,46	-3,46	2,02E-04
259	6,71	102,14	+2,06	-2,06	4,35E-02
271	5,67	25,6	+	-	1,84E-02
272	5,52	33,25	+2,04	-2,04	1,81E-02
279	6,34	42,2	+	-	4,70E-03
281	7,89	41,66	+	-	7,98E-05
282	5,75	41,8	+	-	3,28E-02
284	5,29	41,83	+	-	6,10E-05
294	7,05	54,83	+	-	3,92E-04
299	6,21	93,6	+	-	3,08E-05
300	6,44	94,5	+	-	1,47E-05
307	7,06	40,6	+	-	1,22E-02

**364**	**6,33**	**40,42**	**+3,04**	**-3,04**	**3,29E-02**
