# Supplementary material for: Proteomic analysis of Paracoccidioides brasiliensis complex isolates: Correlation of the levels of differentially expressed proteins with in vivo virulence
Source: PLoS One. 2019 Jul 2;14(7):e0218013. doi: 10.1371/journal.pone.0218013 (PMC6605636; doi:10.1371/journal.pone.0218013)
Supplement: S1 Fig — (A) Paracoccidioides brasiliensis yeast cells were grown for 7 days at 36°C in triplicate on Fava-Netto’s medium, and protein extract was obtained as previously described [1]. Protein concentrations were determined by the Bradford method [2] and 5 μg of each extract were subjected to 1D SDS-PAGE (10%). The molecular masses (in kDa) of standard proteins are given to the left of the gel (BenchMarkTM Protein Ladder, Invitrogen). From left to right: Bot 1/96, Ibiá T1, Ibiá, T2, 262 Uber, T1, Pinguim, Ibiá T2 and Pb18. (B) The completed electrophoresis gel was imaged on an Image Scanner III (GE Healthcare, Uppsala, Sweden) and the comparison was carried out by densitometry measurements of scanned image (8-bit image) along the ROI (arrow) using a 256 grey level scale to determine the average gray value using Adobe Photoshop CC. This region was chosen because it did not present proteins with different abundance levels in the comparative analyzes (2D-GE). (C) Lowest and highest gray values were used to set the ratio between each of the extreme values and the ROI revealing minimum variation across the different samples. (PDF) [file pone.0218013.s003.pdf]

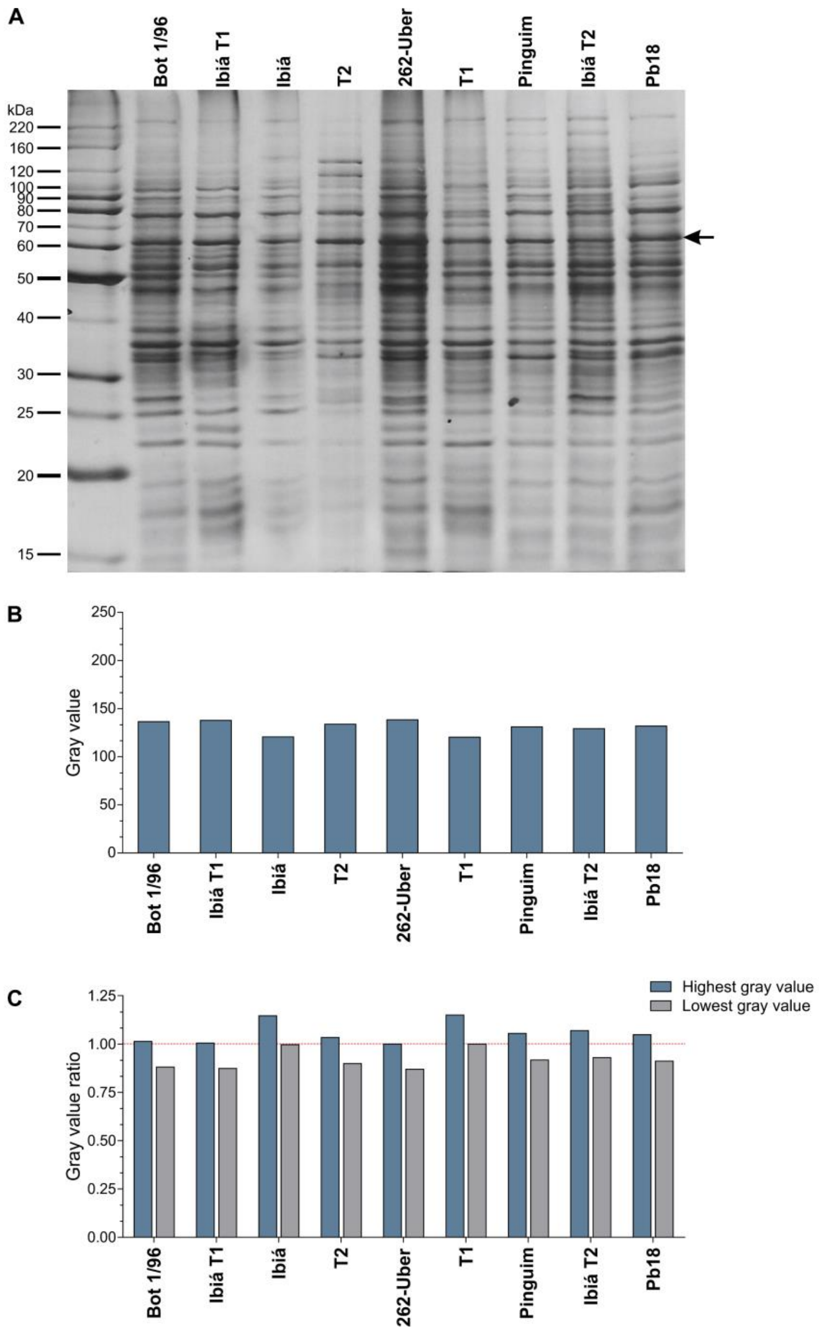

S1 Fig: Protein profile from *Paracoccidioides brasiliensis* strains and densitometry measurements along the region of interest (ROI; arrow). **(A)** *Paracoccidioides brasiliensis* yeast cells were grown for 7 days at 36 °C in triplicate on Fava-Netto's medium, and protein extract was obtained as previously described [1]. Protein concentrations were determined by the Bradford method [2] and 5 µg of each extract were subjected to 1D SDS-PAGE (10%). The molecular masses (in kDa) of standard proteins are given to the left of the gel (BenchMarkTM Protein Ladder, Invitrogen). From left to right: Bot 1/96, Ibiá T1, Ibiá, T2, 262 Uber, T1, Pinguim, Ibiá T2 and Pb18. **(B)** The completed electrophoresis gel was imaged on an Image Scanner III (GE Healthcare,

Uppsala, Sweden) and the comparison was carried out by densitometry measurements of scanned image (8-bit image) along the ROI (arrow) using a 256 grey level scale to determine the average gray value using Adobe Photoshop CC. This region was chosen because it did not present proteins with different abundance levels in the comparative analyzes (2D-GE). **(C)** Lowest and highest gray values were used to set the ratio between each of the extreme values and the ROI revealing minimum variation across the different samples.

References

1. Rodrigues AM, Kubitschek-Barreira PH, Fernandes GF, de Almeida SR, Lopes-Bezerra LM, de Camargo ZP. Immunoproteomic analysis reveals a convergent humoral response signature in the *Sporothrix schenckii* complex. Journal of Proteomics. 2015;115:8-22. doi: 10.1016/j.jprot.2014.11.013. PubMed PMID: 25434489.
2. Bradford MM. A rapid and sensitive method for the quantitation of microgram quantities of protein utilizing the principle of protein-dye binding. Anal Biochem. 1976;72(1–2):248-54. doi: 10.1016/0003-2697(76)90527-3. PubMed PMID: 942051.
